# Reactome graph database: Efficient access to complex pathway data

**DOI:** 10.1371/journal.pcbi.1005968

**Published:** 2018-01-29

**Authors:** Antonio Fabregat, Florian Korninger, Guilherme Viteri, Konstantinos Sidiropoulos, Pablo Marin-Garcia, Peipei Ping, Guanming Wu, Lincoln Stein, Peter D’Eustachio, Henning Hermjakob

**Affiliations:** 1 European Molecular Biology Laboratory, European Bioinformatics Institute (EMBL-EBI), Wellcome Genome Campus, Hinxton, United Kingdom; 2 Open Targets, Wellcome Genome Campus, Hinxton, United Kingdom; 3 Fundación Investigación INCLIVA, Universitat de València, Valencia, Spain; 4 Instituto de Medicina Genomica, Valencia, Spain; 5 NIH BD2K Center of Excellence and Department of Physiology, Medicine and Bioinformatics, University of California, Los Angeles, California, United States of America; 6 Oregon Health and Science University, Portland, Oregon, United States of America; 7 Ontario Institute for Cancer Research, Toronto, Canada; 8 Department of Molecular Genetics, University of Toronto, Toronto, (Canada); 9 NYU Langone Medical Center, New York, New York, United States of America; 10 State Key Laboratory of Proteomics, Beijing Proteome Research Center, Beijing Institute of Radiation Medicine, National Center for Protein Sciences, Beijing, China; Universite de Montreal, CANADA

## Abstract

Reactome is a free, open-source, open-data, curated and peer-reviewed knowledgebase of biomolecular pathways. One of its main priorities is to provide easy and efficient access to its high quality curated data. At present, biological pathway databases typically store their contents in relational databases. This limits access efficiency because there are performance issues associated with queries traversing highly interconnected data. The same data in a graph database can be queried more efficiently. Here we present the rationale behind the adoption of a graph database (Neo4j) as well as the new ContentService (REST API) that provides access to these data. The Neo4j graph database and its query language, Cypher, provide efficient access to the complex Reactome data model, facilitating easy traversal and knowledge discovery. The adoption of this technology greatly improved query efficiency, reducing the average query time by 93%. The web service built on top of the graph database provides programmatic access to Reactome data by object oriented queries, but also supports more complex queries that take advantage of the new underlying graph-based data storage. By adopting graph database technology we are providing a high performance pathway data resource to the community. The Reactome graph database use case shows the power of NoSQL database engines for complex biological data types.

## Author summary

To better support genome analysis, modeling, systems biology and education, we now offer our knowledgebase of biomolecular pathways as a graph database. We have developed a tool to migrate the Reactome content from the relational database used in curation to a graph database during each quarterly release process. The new graph database has two main advantages; higher performance and simpler ways to perform complex queries. Reactome has already adapted its software infrastructure to benefit from this growing in popularity storage technology, significantly improving query efficiency, by reducing the average query time by 93%. We strongly believe that the successful adoption of a graph database by Reactome demonstrates the positive impact this new technology could potentially have in the field and could provide a practical example for other community projects with similar complex data models to move their storage to a graph database while retaining their data models.

This is a *PLOS Computational Biology* Software paper.

## Introduction

Reactome (https://reactome.org) is a free, open-source, open-data, curated and peer-reviewed knowledgebase of biomolecular pathways. Reactome annotates processes in a consistent pathway model to create an online resource for researchers as a core reusable pathway dataset for systems biology approaches. Reactome provides infrastructure and intuitive bioinformatics tools for search, visualisation, interpretation and analysis of pathways [1].

Reactome contains a detailed representation of cellular processes, as an ordered network of molecular reactions, interconnecting terms to form a graph of biological knowledge. Like most biomolecular pathway knowledgebases, Reactome has relied on a relational database to store its content. Although widely used among pathway knowledgebases for data management, relational databases are not always the best fit to deal with today’s performance requirements and increasing data complexity [2, 3]. Relational databases cope well with modeling and storing complex pathway information, but the final product is very likely to contain many intermediate tables to represent many-to-many relationships. As a result, database queries across a network of highly interconnected pathway data are often difficult to formulate and require a high number of join operations, ultimately resulting in degradation of performance and excessive response times.

The Reactome data model naturally forms a large interconnected network that can be seen as a directed graph, which consists of a set of nodes and a collection of directed edges connecting ordered pairs of nodes [4]. Storing Reactome pathway data in its natural form has multiple benefits. Most significantly, it does not require any transformation of data into a flat or denormalised table format. As a result, data can be persisted as originally designed, reducing the complexity of the database and thus allowing a more straightforward access to the Reactome knowledgebase [3].

Here we describe the motivation behind our adoption of a graph database and show how Reactome benefits from this change in the underlying storage technology to overcome the previously mentioned limitations imposed by relational databases. The main target audiences for this manuscript are bioinformatics developers, who might be inspired to apply a graph database in a similar domain, and bioinformaticians involved in pathway analysis, who might benefit from using our graph database directly. While users of the Reactome web interface take advantage of the described gains in performance, features, and stability, the Reactome web interface is described in detail in [1].

## Design and implementation

### The Reactome data model

Reactome uses a frame-based knowledge representation [5]. The data model (https://reactome.org/content/schema) consists of classes (frames) that describe different concepts like reaction or entity. Classes have attributes (slots) that hold properties of the represented class instances, like names or identifiers. The value types contained in the slots can be primitive (string, numbers, or boolean) or references to other class instances. Therefore, knowledge in Reactome is captured as instances of these classes with their associated attributes.

While implementing its relational database, Reactome opted for a physical design that favoured flexibility over performance. Simply put, the relational database incorporated an increased level of abstraction in its physical design resulting in easier adoption of new concepts but at the same time heavily impacting the complexity and execution time of its queries. However, since the graph database natively stores Reactome content in a graph following its model, this trade-off between flexibility and performance is no longer needed.

The Event and PhysicalEntity (PE) classes hold prominent positions in the Reactome model. Events are the building blocks used in Reactome to represent biological processes and are further subclassed into Pathways and ReactionLikeEvents (RLE). RLEs are single-step molecular transformations. RLE includes Reaction among other types like FailedReaction, Polymerisation, Depolymerisation, and BlackBoxEvent. Examples discussed here all involve transformations of the “Reaction” type but all types are handled in the same way with the same results. Pathways are ordered groups of RLEs that together carry out a biological process. PEs are the participants in these events. PE types include SimpleEntity for chemicals, EntityWithAccessionedSequence for proteins, Complex for multi-molecular structures and EntitySet for PEs grouped together on the basis of their shared function.

### Moving from a relational to a graph database

Persistence of a model, like the one described above, can be achieved with flat files, a relational database, or a non-relational database (e.g. a graph database). The selected underlying storage mechanism determines how data are physically stored and accessed. Consequently, each of these options comes with both advantages and disadvantages in terms of performance and scalability. Until recently, Reactome relied on a relational database (MySQL) for both storing its content during curation and accessing it in its production phase. Among the factors that contributed to this decision were that (1) Protégé (http://protege.stanford.edu) was used as the curator tool during Reactome's nascent years with a Perl script processing the Protégé files to store content into a MySQL database, which was modeled according to the Protégé schema, (2) at the time a relational database met Reactome’s needs for data integrity and consistency, and (3) relational databases were well established for biological data whereas graph based solutions were hardly used in the field [6, 7].

It was not until recently that graph databases became a popular technology in different areas of computational biology. Henkel et al. proposed the concept of graph databases for storage and retrieval of computational models of biological systems [7]. Summer et al. developed a Cytoscape application that takes advantage of the Neo4j database to perform server-side analysis of large and complex biological networks [8]. In [9] the authors explored the potential of using a graph database to facilitate data management and analysis to provide biological context to disease-related genes and proteins. Toure et al. developed a Java-based framework that transforms biological pathways represented in SBGN format into the Neo4j graph database, enabling more powerful management and querying of complex biological networks [10]. Balaur et al. demonstrated that advanced exploration of highly connected and comprehensive genome-scale metabolic reconstructions can benefit from an integrated graph representation of the model and associated data [11]. Swainston et al. described biochem4j that enables complex queries by linking a number of widely used chemical, biochemical and biology resources within a graph database [12].

Reactome has gradually introduced a Neo4j graph database (https://neo4j.com/) to store and query its content in the production phase since July 2016 (version 57). Neo4j is an open source, transactional and ACID (Atomicity, Consistency, Isolation, and Durability) compliant graph database [13]. Native graph databases, such as Neo4j, naturally store, manage, analyze, and use data within the context of connections to improve performance and flexibility when handling highly interconnected data compared to that in SQL. Neo4j’s greatest advantage and probably its most defining feature is Cypher: a declarative, pattern matching query language, specifically designed for dealing with graph data structures [14, 15].

**Fig 1:**
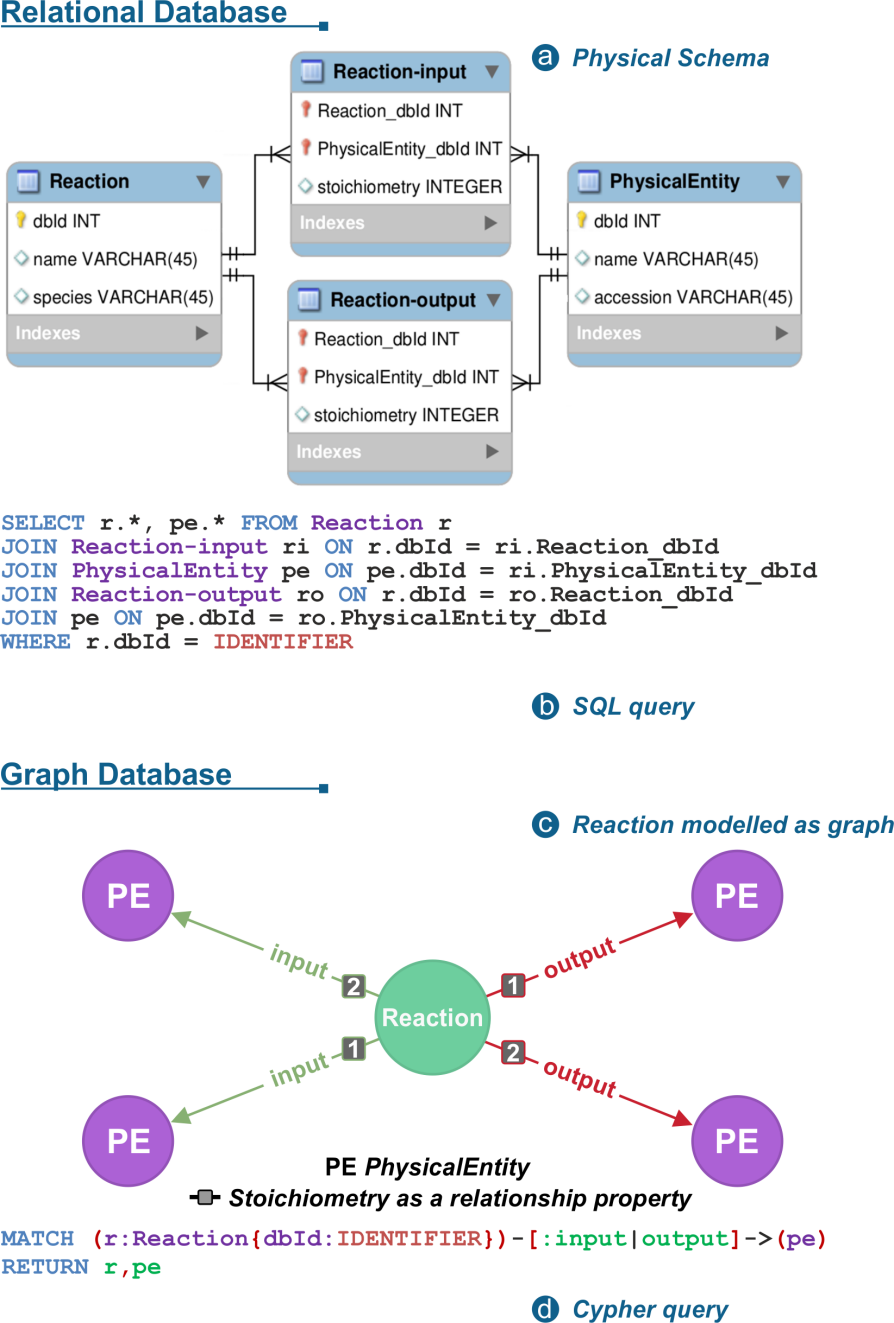
A simplified example where reactions only contain reactants and products represented by the class PhysicalEntity. (a) In the relational use case, two junction tables are required to model these many-to-many relationships (b) SQL query used to retrieve input and output entities of a given reaction where two join operations are needed per junction table. (c) The same reaction modelled as a graph. The reaction (green node) contains named outgoing relationships to corresponding input and output entities (purple nodes). (d) The same query written in Cypher, in a shorter but more intuitive manner.

The Reactome knowledgebase has many use cases, like the one in Fig 1, where the use of a graph model together with a query language like Cypher can greatly improve response times and simplify the code necessary to access the data. For instance, recursively retrieving all reactions of a pathway, retrieving the participants of a reaction or a pathway, deconstructing a complex or a set into its participating molecules, or enumerating the chain of consecutive reactions that lead to the formation of a signalling complex are typical use cases that benefit greatly from traversing the graph version of the Reactome knowledgebase.

Fig 1 provides a simplified example where reactions only contain lists of reactants and products, instances of the PE class. In the relational use case, two junction tables, Reaction-input and Reaction-output, are required to model these many-to-many relationships (Fig 1A). Each junction table contains foreign keys of the Reactions and the associated PEs. The SQL query to retrieve input and output entities of a given reaction requires two join operations per junction table (Fig 1B). In the first stage of its execution, each join operation forms the cartesian product between the tables and, during the filtering process, all rows of the result set that are not of interest are discarded.

The same structure of a reaction with inputs and outputs can be modelled in a simpler way with Neo4j as exemplified by the reaction presented in Fig 1C. The reaction (green node), contains named outgoing relationships to corresponding input and output entities (purple nodes). Taking advantage of Cypher, the same query, can be written in a shorter but more intuitive manner thanks to its ASCII-Art syntax [3] to represent patterns (Fig 1D). The query describes a pattern that includes a Reaction, again identified by its identifier, with its outgoing input and output relationships. Finally, all nodes matching the specified pattern are returned.

Since their introduction in the 1970’s, relational database engines have been optimised to provide efficient execution of SQL queries. This is particularly the case with global queries that aggregate large amounts of data without the need to perform any traversal operations. However, Reactome data contain many relationships, like those illustrated in Fig 1, and thus many join tables, so queries generally require traversal operations, a computational intensive task that tends to result in poor performance compared to graph databases [16]. To address this issue and improve query performance, some resources have created redundant denormalised copies of their relational database [17, 18, 19]. Nowadays, graph databases, such as Neo4j, offer a more appropriate alternative for cases of highly interconnected data.

### The new graph database ecosystem

**Fig 2:**
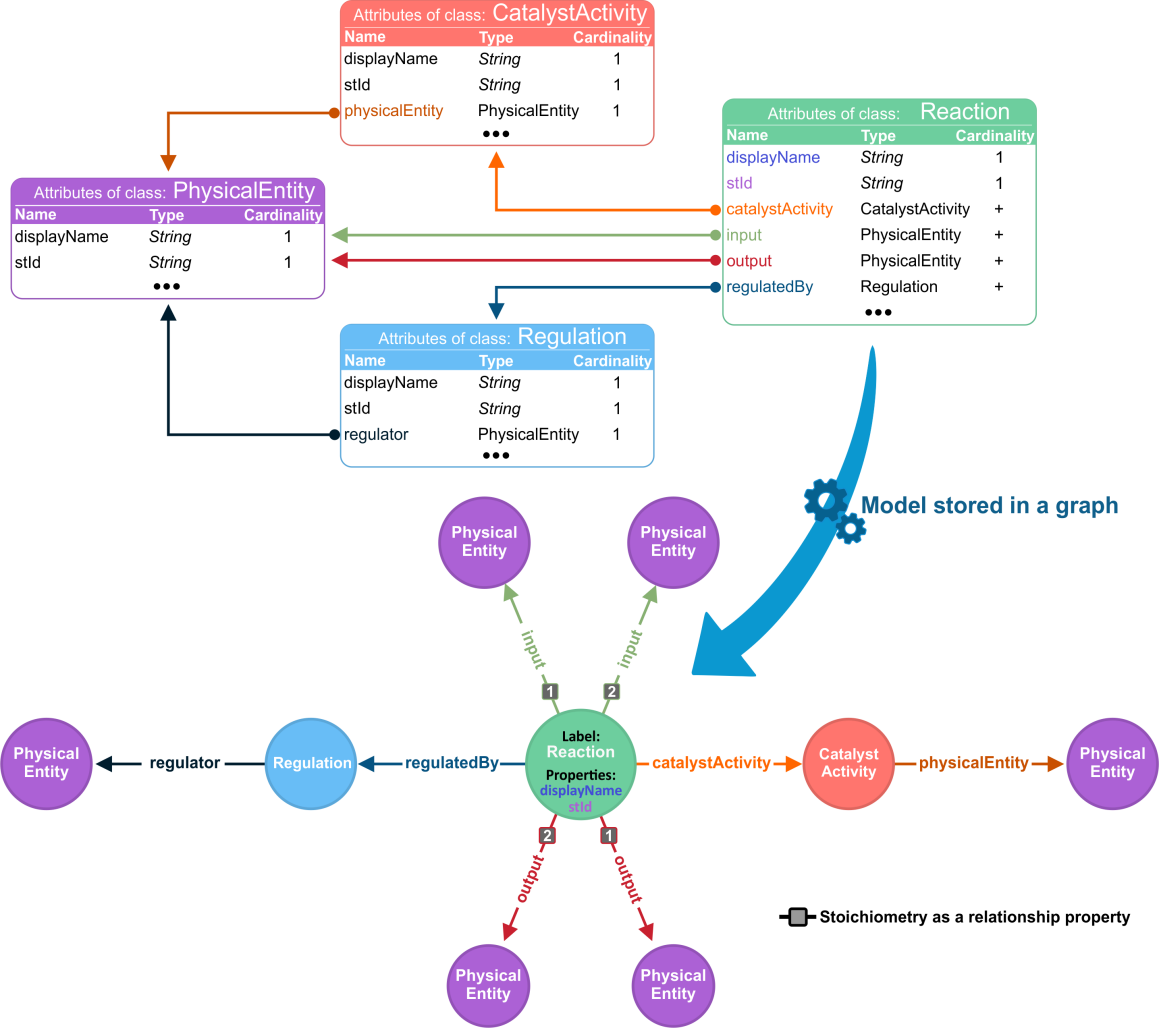
Representation of the content migration. The example shows a Reaction class reduced to its inputs, outputs, catalyst and regulators. A model class instance is converted to a graph database node where (1) slots with primitive value types become node properties and (2) slots allocating instances of another class become relationships.

The graph database batch importer (https://github.com/reactome/graph-importer) was developed to migrate the content from the relational database used in curation, to a graph database during each quarterly release process. Although the underlying data storage was changed, the original data model used by MySQL was kept the same. The conversion was done following a depth-first approach starting from the top level pathways and traversing all the content, ensuring that each object is processed only once during the conversion. Every object constitutes a node in the graph and the edges that connect the nodes correspond to the names of the slots as defined in the domain model (Fig 2). As a result, a Neo4j graph database is generated and contains all the Reactome data. It can be directly used for third parties in order to use Cypher to retrieve the target data.

A number of integrity tests have been put in place to ensure that both the graph and relational database have the same content after conversion. These tests are part of the graph-core and they are executed after migrating the relational database to the graph database to ensure that the data has been properly stored. The tests include checks to verify: that the number of top level pathways present in the graph database corresponds to the number of those present in the relational database; that a given pathway in the graph database has the same ancestors as its counterpart in the relational database; that the content of a given complex is the same in both databases.

**Fig 3:**
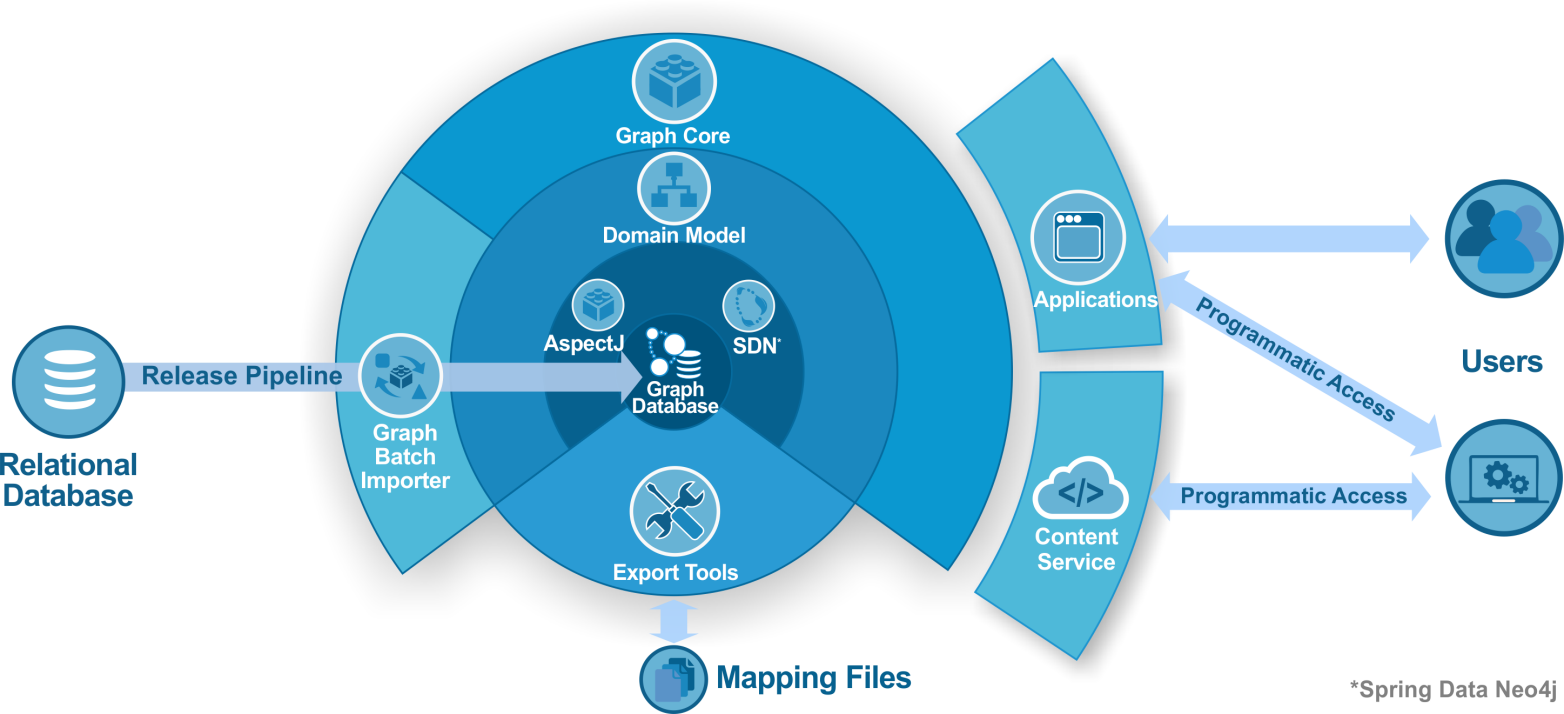
A schematic diagram of the new ecosystem. The relational database is converted to a graph database via the batch importer that relies on the Domain Model. Spring Data Neo4j and AspectJ are two main pillars for the graph-core, which also rests on the Domain Model. Users access services or use tools that make direct use of the graph-core as a library that eliminates the code boilerplate for data retrieval and offers a data persistency mechanism. Finally, export tools take advantage of Cypher to generate flat mapping files.

Fig 3 presents a schematic illustration of the new Reactome graph database ecosystem. A library called graph-core (https://github.com/reactome/graph-core) was developed on top of the graph database to serve as a data access layer. The aim of the library is to provide easy access and data persistence as well as to reduce the boilerplate code in third party projects that require accessing and traversing Reactome content. The graph-core uses Spring Data Neo4j (SDN) [20] to access the graph content and AspectJ to enable lazy loading [21]. Lazy loading commonly refers to a design pattern, that postpones the retrieval of object attributes until the point at which they are needed. In our case, AspectJ weaver is used to intercept the getter methods and run specific code to silently retrieve more data when needed.

The ContentService (https://reactome.org/ContentService) is a REST based web service [22], built on top of the graph-core, to provide programmatic access to the Graph Database for third party developers (https://github.com/reactome/content-service). Implemented on top of Spring MVC (https://spring.io/), the ContentService utilises the graph-core library and is fully documented with Open API (https://www.openapis.org/).

## Results and discussion

Among its main advantages, this new solution is faster and less computationally intensive than the previous one based on the relational database. Performing queries against the graph database constitutes a more scalable approach, resulting in higher throughput and, ultimately, to a more robust ContentService able to cope with an always increasing number of requests. Additionally, the resulting product is easier to maintain as most new methods can be added by simply writing the respective Cypher queries, avoiding writing complex algorithms in a given programming language (Fig 1B).

**Fig 4:**
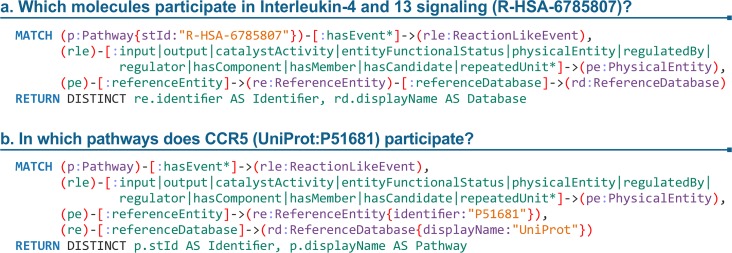
Examples of frequent use cases that can be answered using Cypher queries. a) Retrieving the participating molecules for “Interleukin-4 and 13 signalling” pathway. b) Retrieving the pathways in which CCR5 participates.

The use cases above are available as methods in the ContentService API (https://reactome.org/ContentService/). Fig 4 emphasises how queries of Reactome data have been simplified by the adoption of the graph database. The query in Fig 4A shows how to retrieve the participating molecules for a pathway. The reverse query, identifying pathways where a molecule participates, is shown in Fig 4B, which follows a similar pattern to Fig 4A, but fixes the end-bound and leaves the upper-side open for traversing results. Based on feedback provided by people contacting our help desk (help@reactome.org) and attending our training sessions, the new way of querying Reactome is easy and intuitive to learn, and researchers, who are interested in performing queries against Reactome data, can learn to write them in Cypher in a relatively short amount of time.

To assess the improvement we designed a set of stress tests to measure the impact of adopting the graph database in Reactome. All stress tests were executed on a standard laptop featuring an Intel Core i7 at 2.6 GHz, 16 GB of DDR3 memory at 1,600 MHz, and 256 GB of flash storage. The tests do not aim to compare the two storage technologies (MySQL and Neo4j) but instead their usage by Reactome. The stress tests were run against the web services build on top of each storage technology and included two scenarios: (1) simulation of one user sequentially querying 5,000 reactions for *Homo sapiens* and (2) simulating an increasing set of users simultaneously performing the previous task. In each case the resulting data for every reaction had to be marshalled as an instance of the correspondent model class. The test comprised four executions; two against the previous web service running on top of the relational database and the other two accessing the new web service running on top of the graph database through the newly created graph-core library (https://github.com/reactome/graph-core). The reactions were accessed in a sequential fashion to ensure that caching did not provide any sort of advantage for any of the approaches, because a queried object would never be retrieved again in the same test. It should be mentioned that prior to any stress test’s execution, both Neo4j and MySQL databases were configured to allocate 50% of the available physical memory (8GB).

**Fig 5:**
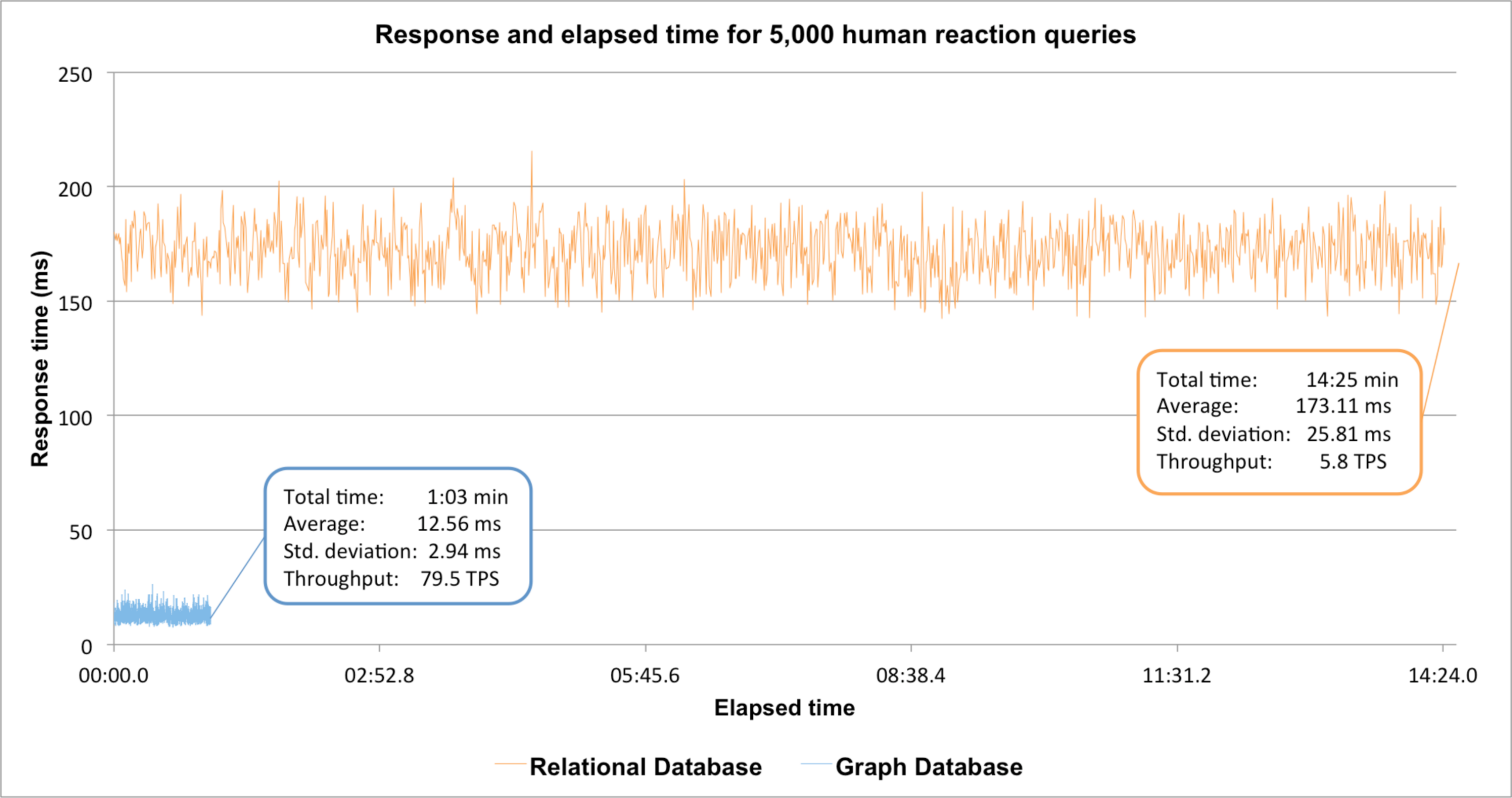
Comparison of the response and elapsed time for one user sequentially retrieving 5,000 reaction instances from the graph and relational databases (blue and orange respectively). The graph database software ecosystem achieved a 93% average improvement in performance compared to that of the relational database.

As illustrated in Fig 5, querying the data stored in the relational database resulted in significantly longer response times. In particular, in the case of the relational implementation of the Reactome knowledgebase the average query time was 173.11 ms (±25.81) while in the case of the graph implementation, the average response time dropped to 12.56 ms (±2.94),a 93% reduction in the average query time. The new implementation supported higher throughput, in terms of transactions per second (TPS), reaching 79.5 TPS compared to 5.8 TPS. As a result of this boost in performance, all 5,000 queries to the graph database were performed in 63 seconds while the relational implementation required more than 14 minutes for the same task.

**Fig 6:**
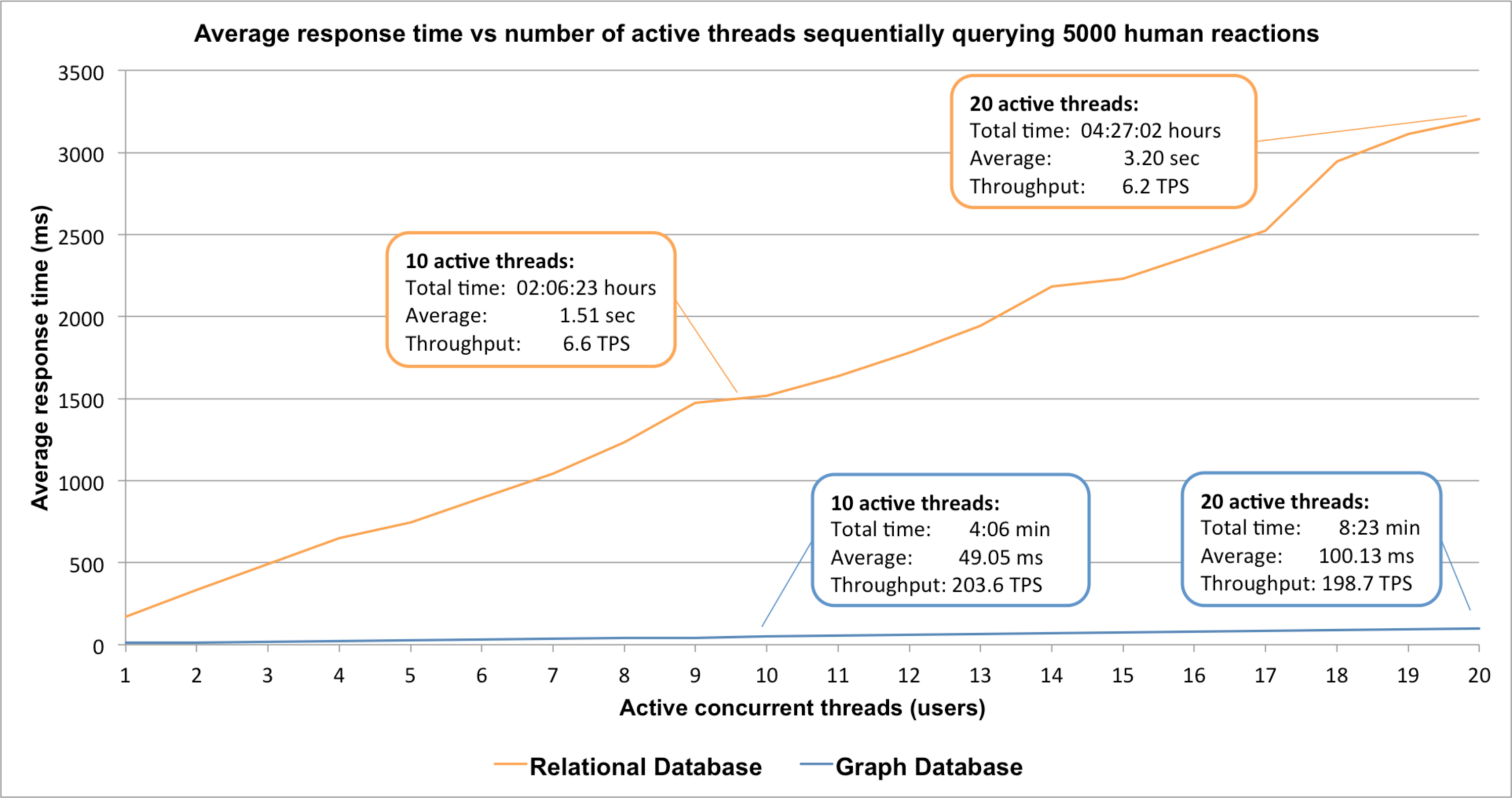
Response time versus an increasing set of users simultaneously performing queries for 5,000 reaction instances. Starting with one and scaling up to 20 concurrent users, the relational database performance drops while the graph database keeps a low response time and a good throughput as the number of active threads increases.

A second stress test simulated a more realistic scenario where multiple users perform concurrent database queries (Fig 6). Once again, querying the Reactome knowledgebase in its relational implementation resulted in significantly longer response times. For instance, in case of 10 concurrent threads performing queries to the relational implementation of the Reactome knowledgebase the average response time was 1,516 ms while in the case of the graph implementation, the average response time dropped to 49.05 ms. In addition, the new implementation achieved higher throughput reaching 203.6 TPS compared to 6.6 TPS. Consequently, the graph implementation of Reactome provides higher scalability enabling Reactome to handle larger volumes of user requests.

**Fig 7:**
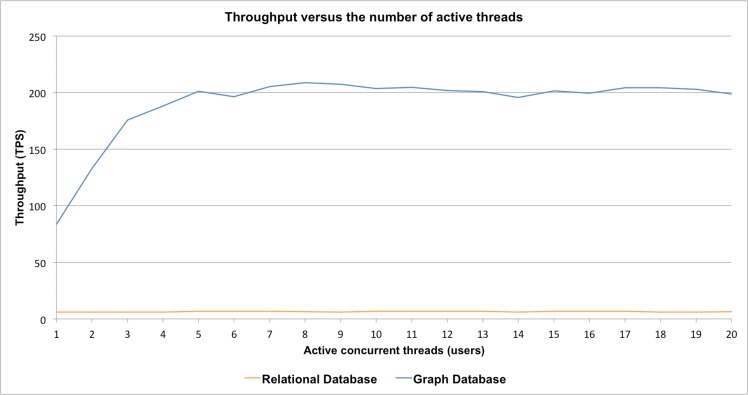
Throughput measured in transactions per second, versus the number of users concurrently performing queries for 5,000 reaction instances in *Homo sapiens*.

Fig 7 presents a comparison between the throughputs achieved by both systems against the number of users performing concurrent queries. The graph implementation achieved a higher number of transactions per second that reached a plateau after the point where the number of active threads becomes equal to the available processor cores; in this case 4. On the other hand, the measured throughput in case of the relational implementation is stable and does not seem to take advantage of any concurrency.

**Fig 8:**
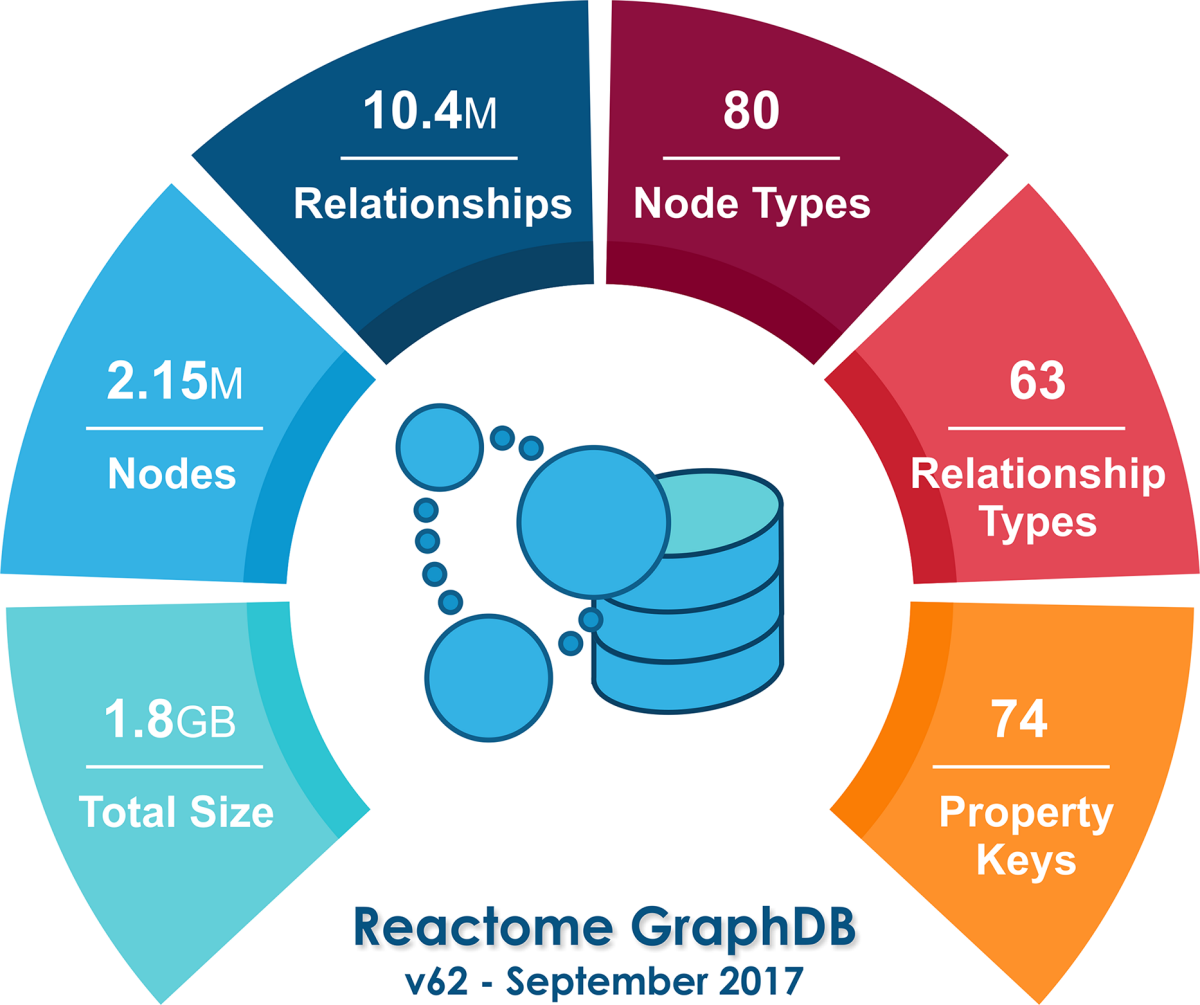
The Reactome graph database in numbers.

Many users choose to download the Reactome graph database and access the data through Cypher queries directly in their computers. Our usage statistics show that a growing number of users have downloaded the Reactome graph database and, based on the questions gathered by our help desk service, we believe that they have used it to perform local queries against the complete Reactome knowledgebase. In particular, during the first year that Reactome provided the graph database, there were 2,385 downloads by 912 unique users. 118 of those users downloaded the graph database after each data release. It is worth mentioning that during writing of this manuscript, the size of the Reactome relational database in its current data release (v62) is around 2.0GB while the size of the graph database is approximately 1.8GB. Fig 8 provides a summary of the graph database.

With a tool so powerful at managing highly connected data sets and complex queries at our disposal, Reactome is providing faster and more stable services to researchers around the world. In the near future, Reactome plans to upgrade its services and leverage the full potential of Cypher in order to provide answers to questions that require diving deeper into our data. In particular, the integration of a graph database lowers the complexity of problems that require traversing of our knowledgebase, such as identifying causal interactions or revealing all possible paths between two molecules.

Future development in Reactome is not likely to be affected by the fact that Neo4j is by nature schema-less, mainly because the rigid schema of our relational database with all the applied constraints is used to ensure data consistency during the curation phase. Currently, data are migrated to Neo4j during each quarterly release process and are used to speed up queries in production.

In conclusion, through the adoption of the Neo4j graph database, and by harnessing the power of its query language, Reactome provides efficient access to its pathway knowledgebase. As a result of this shift in the underlying data storage technology, the average query time has been reduced up to 93%. In addition, the graph-core library and the ContentService leverage these benefits of this shift and can be used by third party applications to efficiently access Reactome.

Reactome’s successful use case constitutes a strong argument in favour of the positive impact this new technology can have in the field. By following Reactome’s use case, other community projects with similar complex models could benefit from moving their storage to a graph database while keeping their data model. While we have demonstrated the major impact of moving the Reactome public database to a graph database in terms of usability, stability, and response time, we think this is only a milestone in the growing ecosystem of network-oriented biomolecular data resources that will enable entirely new functionalities through moving to modern database technology that better reflects the graph-like structure of their source data. While we will work directly with internal and external resources to move along that path, we would also like to invite the community to use the open data Reactome graph database to develop their own novel uses of Reactome data.

## Availability and future directions

The Reactome graph database is freely available at: https://reactome.org/dev/graph-database. The API for the ContentService is available at https://reactome.org/ContentService with documentation and tutorials available at: https://reactome.org/dev/content-service. The source code, in Java, is freely available at: https://github.com/reactome (See the graph-core, graph-importer and content-service repositories).

Future development will focus on updating the version of SDN and integrating interaction data from IntAct (http://www.ebi.ac.uk/intact/) directly to the Reactome graph database.
